# Practical Notes and Formulæ

**Published:** 1879-10-20

**Authors:** 


### PRACTICAL NOTES AND FORMULÆ.

**Cholera Infantum.**—Dr. Charles H. Avery, of New York, in Medical Record, writes that he has adopted the following treatment in cholera infantum: He first directs that a poultice be made as follows, and applied over the stomach.

Take of pounded cloves, cinnamon, and ginger, each one teaspoonful, add a small quantity of flour, and then moisten the whole with brandy. Spread the mixture on and cover with thin flannel, and so fasten it that it will be kept in position. Occasionally moisten the poultice with brandy, which can be done without removing it.

One teaspoonful of the following mixture is then ordered every two hours for children over three months old:

| | | | |
|---|---|---|---|
| ℞ | Acid carbol | gr. iv. | |
| | Spts. vini | gtt. xxiv. | |
| | Aqua menth pip | ℥ iss. | |
| | Mucil. acac | ʒ vi. | |
| | Syr. papaver | ʒ vi. | |
| | Tinct. opii | gtt. x. | M. |

As a rule the vomiting ceases before the hour arrives for the administration of the third dose; frequently before the second dose is given.

The passages from the bowels are not arrested by the medicine, but within twenty-four or forty-eight hours they begin to change in character, soon diminish in frequency, and afterwards cease altogether. The diet of the child is restricted to barley-water and milk. If it is a nursing child, barley-water is administered before it is allowed to take the breast.

If the vomiting is severe, the child is not allowed to take anything, except the medicine, for three hours.

If there is marked evidence of acidity of the digestive tract, teaspoonful doses of the following mixture are given every ten or fifteen minutes for two or three hours.

| | | | |
|---|---|---|---|
| ℞ | Mistura cretæ | ℥ ij. | |
| | Syr. rhei | ℥ i. | M. |

To this he sometimes adds fifteen grains of hydrarg. cum creta.

As a substitute for the above antacid mixture, he sometimes gives ten grains of subnitrate of bismuth, and five grains of pepsin three times a day.

The leading features of the plan which he recommends are: the spice poultice, the barley-water and milk diet, and the medicines according to the first prescription.—*Canada Lancet.*

**Calomel.**—A correspondent of St. Louis Courier of Medicine says: "The alterability of calomel has attracted the attention of several investigators. Jolly has found that light, dilute hydrochloric acid, chloride of sodium, magnesium and calcium, and carbonate of sodium, all have a tendency to change calomel into corrosive sublimate and metallic mercury. Experiments in the same direction made by Frederick M. Corwin have given similar results, although they disagree in some respects from those obtained by Mr. Jolly."

**How to Prevent Mammary Abscess.**—It is not well to wait until there are marked evidences of inflammation. A good rule is to ask the patient if the breast feels heavy and drag so much to the side as to impede the movements of her arms, and particularly to prevent her from bringing her arms close to the side of the body. This being the case, take two or three broad strips of plaster (which, if good, will not need heating), direct the nurse to lift the breast gently toward the median line of the body upon the sternum, then apply one strip on the lower half of the breast, the other on the upper half, reaching from one axilla to the other. Other auxiliary strips may be applied according to the size of the breasts and effectiveness of the first two. When the breasts have become "caked," and the patient is so uncomfortable as to be unable to sleep, this procedure will generally be followed by immediate relief from discomfort, and the milk will oftentimes flow from the nipple in streams.—*Med. Herald.*

**Dropsical Affections.**—An excellent remedy in dropsical affections:

℞ Elaterii ........................................ gr. j.
Ext. colocynthidis comp .......................... gr. xxxvj.
Ext. Hyoscyam .................................... gr. xij .M.
Ft. Mas. in pill xij, dividenda.

Dose, one pill every day for two or three days; after which one every thirty-six hours.

**Rheumatic Iritis.**—Dr. Galezowsky, in a note to the Academie de Medecine, reports the result obtained by the use of the salicylate of soda used internally in this disease, as follows: "In eighteen cases an amendment in all the symptoms was obtained, often in three or four days, in the same patients, who, prior to its use, had been under treatment with similar symptoms for a month or six weeks; the most remarkable feature being the immediate disappearance of the pain and redness, and later, the subsidence of the exudation.

"Excellent results have also been obtained in rheumatic inflammations of the sclerotic; this was especially noticed in eighteen cases of scleritis and sclero-keratitis, where other treatment had been of no avail after several months, the salicylate producing a cure in from one to six weeks."—*N. Y. Med. Journal.*

**Painful Diarhœa, etc.**—The following is an infallible remedy for excessive and painful diarrhœa, dysentery, etc.

℞ Tinct. catechu ..........................................
Tinct. opii ...................................... aa ℥j.
Syrup rhei aromat .............................. partes equal.

M. Dose, half to teaspoonful every three or four hours.

**Salicylic Acid in Ear Diseases.**—Dr. Bezold finds that salicylic acid succeeds well as a parasiticid and rapidly destroys otomycosis.

Perforations of the membrana tympani cicatrize very rapidly when treated with injections of this drug.—Dr. MOORE, in *N. Y. Medical Journal.*

**Feted Discharges from Tooth Abscess Opening into the Antrum.**—This form of ozena, says Naphey, is cured, as a rule, by extracting the tooth. If this does not succeed injections are to be made through the tooth alveolus. A good one to commence for a day or two with is a solution of the permanganate of potash.

℞. Permanganate potash........................... ʒ ss.

Aquæ.................................................... ℥ viij.

This can be employed three times a day, after which one of the following can be employed:

℞. Tinc. iodinii.....................................

Glycerinæ ad............................................aa ℥ j.

Acidi tanici............................................... ʒ j.

Aquæ coloniæ........................................... ℥ j.

Aquæ distil............................................... ℥ iij.

or,

℞. Tinc. capsici compositæ............................ ℥ ss.

Aquæ rosae ............................................... ℥ viij.

**Rapid Cure of Coryza.**—Rodolfe claims that a fresh case of coryza may be cut short within an hour by chewing one or two dried eucalyptus leaves and swallowing the exceedingly bitter and aromatic saliva. In chronic cases it has no effect.—*St. Petersburger Med. Wochenschrift*, No. 34, 1879.

**Gonorrhœa.**—Dr. Doan, in Medical Brief, suggests the following treatment:

First. Regulate the diet by excluding meats of all kinds, fat, tea, coffee, alcoholic and malt liquors, tobacco in all forms.

Second. Give a purge of a simple character in the afternoon of the day when case is first seen, and after that day if required by constipation.

Third. Bathe the penis and hips in as warm water as the patient can endure three times a day.

Fourth. Render the urine, alkaline and sedative by

R. Potash bi carb................................ 2 drachms.
Tr. hyoscyamus................................ 2 ounces.
Syr. simplicis.................................. 1½ drachms.
Water............................................... 8 ounces.

M. Sig. Tablespoonful four times a day in water.

Fifth. Support the scrotum by a T bandage or suspensory bag, to prevent statis of blood in the scrotum and epididymytis.

**Laxative Bread.**—Mr. W. H. Taylor, in the Lancet, says that he has bread prepared as follows, and found it most useful in constipation and as a laxative in piles: Coarse Scotch oat meal, whole wheaten flour, coarse ordinary flour, of each equal parts. The bread can be lightened by yeast, or, to a two pound loaf, one tablespoon of baking powder, made of four ounces of bi-carbonate of soda, three ounces of tartaric acid, one pound of ordinary flour, rubbed well together and kept dry in a tin or well corked bottle. The bread keeps well, and a two pound loaf will be sufficient for a week, baking a portion once or twice a day in conjunction with ordinary bread.—*Hospital Gazette.*

**Pertussis.**—Dr. R. Otto, believing whooping-cough to be due to the presence of a vegetable parasite, employed an inhalation of a two per cent. solution salicylic acid, in an epidemic which occurred at Lisetz in Livonia, in August, 1876. The treatment was applied in seven cases, commencing with the convulsive stage. The number of paroxysms was rapidly diminished in all the cases, the best results being obtained in children in rooms uniformly heated. The number of cases being so few, show what can be done, and call for further trials in this disease.

**Burns.**—For burns of third and fourth degree :

℞ Ung. petrolii,

Cerat resinæ ........................................... aa ℥ ss.

Morph. sulph.............................................. gr. ii.

Acidi carbolici............................................. gtt. xx. M

Sig. Keep surface well covered.

**Nursing Sore Mouth.**—Dr. Sanders, in Medical Brief, says: I notice several remedies for nursing sore mouth, and would call attention to the following treatment which I saw recommended some years ago, and which I have used several times with complete success. It is iodide potass. in one grain doses three times a day. No local treatment is used, nor do I think, from my experience with strictly local applications, that they are worth a trial.

**Salicylic Acid as an Anti-Scorbutic.**—Dr. L. G. Lincecum, of Texas, would call the attention of the profession to the use of salicylic acid as an anti-scorbutic. I have used it in seven cases with perfect success. Given internally one gramme, daily, in solution ; with carbolated water as an external application. I hope the profession will give it a trial in all scorbutic troubles.

**Cholera Morbus.**—This is a faithful remedy for cholera morbus, colics, etc.

℞ Spts. ether., comp.,
Spirit ammonia aromat,
Chloroformi, equal parts. M.

Dose, teaspoonful every half-hour or every hour, as occasion demands, until relieved.

The first dose will usually suffice.

**Hemorrhage from the Nose.**—Hemorrhage from the nose almost invariably proceeds from twigs of the facial artery which are supplied to the structures lining the interior nares, and may almost always be arrested by compressing the alæ firmly between the thumb and finger. Finding a disposition of the bleeding to return, illuminate passage and apply a plug of cotton wool wet with some styptic lotion directly to the bleeding surface.—*Medical Herald.*

